# A single-center analysis of Henoch-Schonlein purpura nephritis with nephrotic proteinuria in children

**DOI:** 10.1186/s12969-017-0146-4

**Published:** 2017-03-04

**Authors:** Dan Feng, Wen-Yan Huang, Sheng Hao, Xiao-Ling Niu, Ping Wang, Ying Wu, Guang-Hua Zhu

**Affiliations:** Department of Nephrology and Rheumatology, Shanghai Children’s Hospital, Shanghai Jiao Tong University, 355 Luding Road, Shanghai, 200062 People’s Republic of China

**Keywords:** Henoch-Schonlein Purpura nephritis, Nephrotic proteinuria, Clinical features, Prognosis, Children

## Abstract

**Background:**

In children with Henoch-Schonlein purpura nephritis (HSPN), the degree of proteinuria has been proven to be not only a sign of kidney damage, but also an accelerator of kidney disease progression. Nephrotic proteinuria at disease onset has been proposed as a predictor of a poor renal outcome. This study aims to assess the clinical and pathological features of HSPN with nephrotic proteinuria in a single center.

**Methods:**

One hundred thirty-seven patients with HSPN who visited Shanghai Children’s Hospital from January 2009 to December 2013 were retrospectively reviewed. The patients were divided into 2 groups based on the 24-h urinary protein levels: nephrotic proteinuria group (NP group: 24-h urinary protein ≥50 mg/kg) and non-nephrotic proteinuria group (NNP group: 24-h urinary protein <50 mg/kg). In addition, data regarding their sex, age, clinical features, renal pathology, and prognosis were collected.

**Results:**

(1) There were 34 boys and 20 girls in the NP group with a mean age of 8.39 ± 2.85 years. The peak age of incidence was 6 to 11 years (72.22%). (2) There were 8 cases (14.81%) with joint symptoms and 9 cases (16.67%) with gastrointestinal symptoms in the NP group. According to the analysis of the laboratory test results, the serum albumin and IgG levels of the NP group were significantly lower than that of the NNP group (35.04 ± 8.45 in the NP group vs. 41.55 ± 4.46 in the NNP group, *P* < 0.0001; 7.68 ± 3.12 in the NP group vs. 9.53 ± 2.74 in the NNP group, *P* < 0.001, respectively); their blood urea nitrogen and cystatin C levels increased significantly (*P* < 0.05). (3) The majority of the pathological changes in the NP group were above the International Study of Kidney Disease in Children (ISKDC) grade III (62.97%). The NP group patients with tubulointerstitial injurie with grade 2 and above (50%) were prioritized. Immune complex deposition in the NP group was dominated by IgA. (4) The prognosis of the NP group was in complete remission (A), and their cases did not develop into end-stage renal disease; their prognosis was also associated with clinical classification (*P* < 0.01) but was not related to pathologic grading and tubulointerstitial injury (*P* > 0.05).

**Conclusion:**

The serum albumin and IgG levels of the NP group were significantly lower; however, their blood urea nitrogen and cystatin C levels were higher. The ISKDC grades were mainly above grade III. The prognosis of the NP group was associated with clinical classification and improved after a timely and early treatment.

## Background

The long-term outcomes of Henoch-Schonlein purpura (HSP), a common type of systemic vasculitis in children, depend on its renal involvement [1]; such condition is referred to as HSP nephritis (HSPN). A previous epidemiological survey showed that the incidence of HSPN has gradually risen in recent years. Furthermore, the degree of proteinuria has been proven to be not only a sign of kidney damage but also an accelerator of kidney disease progression [2]. Nephrotic proteinuria (NP) in HSPN often yields a poor prognosis. Therefore, it is important to detect NP in HSPN cases. However, no previous studies have investigated the relationship between the outcomes and clinical features of NP in HSPN. On this basis, children with HSPN who presented with NP were retrospectively studied to define the risk factors of poor prognosis accurately and provide a foundation for clinical treatment.

## Methods

One hundred thirty-seven children diagnosed with HSPN who were admitted to Shanghai Children’s Hospital between January 2009 and December 2013 were retrospectively analyzed.

A retrospective analysis of all the data of the selected patients was performed by reviewing their medical files. The patients were divided into the NP group (24-h urinary protein ≥50 mg/kg) and non-NP (NNP) group (24-h urinary protein <50 mg/kg) based on their 24-h urinary protein levels. General information, which included age, sex, height, and weight, and symptoms, which included the frequency of skin, joint, and gastrointestinal involvements of the 137 patients were evaluated. The levels of albumin (Alb), blood urea nitrogen (BUN), serum creatinine (Scr), uric acid (UA), cystatin C (Cys-C), prothrombin time (PT), activated partial prothrombin time (APTT), thrombin time (TT), fibrinogen (FIB), fibrinogen degradation product (FDP), d-dimer, antithrombin III (AT-III), platelet (PLT), estimated glomerular filtration rate (eGFR), immunoglobulins (IgA, IgG, IgM, and IgE), cellular immunity, and complements were also evaluated.

The clinical spectrum of HSPN ranges from isolated microscopic hematuria to nephrotic syndrome, rapidly progressive glomerulonephritis, and renal failure.

The histological grades of the renal biopsy were graded in accordance with the International Study of Kidney Disease in Children (ISKDC) classification [3] as follows: grade I, minor glomerular abnormalities; grade II, pure mesangial proliferation; grade III, minor glomerular abnormalities or mesangial proliferation, with crescents/segmental lesions (sclerosis, adhesion, thrombosis, and necrosis) in <50% of the glomeruli; grade IV, same as grade III but with crescents/segmental lesions in 50–75% of the glomeruli; grade V, same as grade III but with crescents/segmental lesions in >75% of the glomeruli; and grade VI, membranoproliferative-like lesions and tubulointerstitial lesions (interstitial inflammation or fibrosis and tubular loss); the vascular component was graded as follows: grade 1, essentially normal; grade 2, <25%; grade 3, 25% to 50%; and grade 4, >50% [4].

Long-term outcomes were classified as follows [3]: A, normal (no hypertension and normal physical examination, urine, and renal function); B, minor urinary abnormalities (normal physical examination with microscopic hematuria or mild proteinuria); C, active renal disease (Hypertension, heavy proteinuria, or eGFR of <90 mL/min/1.73 m^2^); and D, end-stage renal disease (ESRD) or death.

### Statistical analyses

All statistical analyses were performed using the SAS software. Quantitative variables were reported as mean ± standard deviation. In addition, the Spearman rank correlation test was used in the analysis of the correlations. *P* < 0.05 was considered statistically significant.

## Results

### Clinical features

**Fig. 1:**
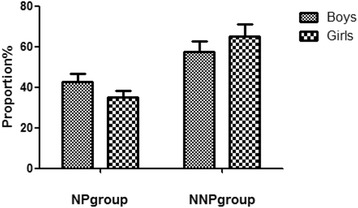
Sex distribution of the NP and NNP groups. The NP group had 34 boys (42.50% of the total boys) and 20 girls (35.09% of the total girls). The NNP group had 46 boys (57.50% of the total boys) and 37 girls (64.91% of the total girls). NP, nephrotic proteinuria; NNP, non-NP

This study included 137 patients, with 80 boys and 57 girls, making the boy to girl ratio 1.40:1. The NP group had 54 patients (34 boys, 20 girls; boy:girl = 1.70:1), and the NNP group had 83 patients (46 boys, 37 girls; boy:girl = 1.24:1) (Fig. 1). The statistical data showed that there were significantly more boys than girls; however, the NP group and NNP group exhibited no sex differences (*P* > 0.05) (Fig. 1).

**Fig. 2:**
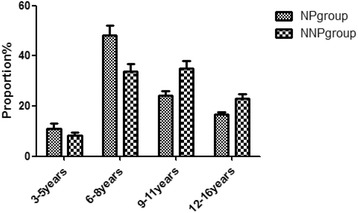
Age distribution of the NP and NNP groups. The youngest patient was 3 years old, and the oldest patient was 16 years old. We divided the patients into 4 groups according to their age. NP, nephrotic proteinuria; NNP, non-NP

The age of all patients ranged from 3 to 16 years, and the mean age was 8.50 ± 2.91 years. The mean age of the NP group was 8.39 ± 2.85 years, and the peak age was 6–11 years (72.22%), especially the range of 6–8 years old. The mean age of the NNP group was 9.21 ± 2.94 years. These two sets of data exhibited no statistically significant differences (Fig. 2).

**Fig. 3:**
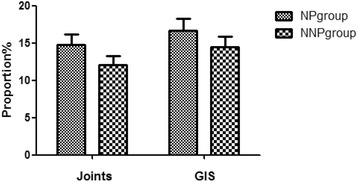
The clinical features of the NP and NNP groups. the NP group had more patients with joint (14.81%) and gastrointestinal (16.67%) symptoms

There was a clear specificity of HSPN with NP clinical manifestations. There were 8 cases (14.81%) with joint symptoms and 9 cases (16.67%) with gastrointestinal symptoms in the NP group; there were 10 cases (12.04%) with joint symptoms and 12 cases (14.46%) with gastrointestinal symptoms in the NNP group. Compared with the NNP group, the NP group had more patients with joint and gastrointestinal symptoms. However, no statistically significant difference was found by the comparison (*P* > 0.05). Data are shown in Fig. 3.

### Laboratory examination

To further investigate the characteristics and significance of NP in HSPN, the laboratory test results of all patients with HSPN were analyzed. The NP group had lower blood Alb and higher BUN and Cys-C levels (*P* < 0.05). The results are shown in Table 1.

**Table 1:** Comparison of laboratory data at one set in NP and NNP

Laboratory data	NP group *x* − ±s	NNP group *x* − ±s	*t*-test/Wilcoxon	*P*
PLT	342.02 (91.55)	317.98 (97.18)	−1.57	0.12
AT-3	165.8 (50.18)	178.2 (52.51)	1.36	0.18
PT	11.14 (0.86)	11.29 (0.90)	1.01	0.31
APTT	25.65 (4.41)	26.69 (4.61)	1.38	0.17
FIB	2.76 (0.92)	2.58 (0.74)	−1.35	0.18
FDP	1.60 (2.60)	2.20 (4.70)	−1.74	0.08
d-dimer	0.30 (0.60)	0.20 (0.35)	1.59	0.11
eGFR	175.10 (58.31)	184.30 (41.36)	1.07	0.29
Alb	35.04 (8.45)	41.55 (4.46)	5.47	<0.0001
BUN	5.81 (3.32)	4.72 (3.25)	−2.03	0.04
Cr	43.82 (24.83)	40.11 (16.59)	−1.03	0.31
UA	308.30 (91.86)	275.10 (78.93)	−1.68	0.09
Cys-c	1.19 (0.57)	0.85 (0.26)	−2.76	0.01

### Immunologic analysis

At the beginning of the process of the illness, there were obvious abnormalities in the humoral and cellular immunities in the children diagnosed with HSPN. Immunological index included blood immunoglobulin levels (IgA, IgG, IgM, and IgE) and lymphocyte subgroup analysis (CD3+, CD4+, CD8+, CD16 + 56+/CD3+, CD19+, and CD4+/CD8+). Further, the level of complements (CH50, C3, and C4) was analyzed. The statistical analysis showed that the level of blood IgG of the NP group was lower than that of the NNP group (*P* < 0.05). The results are shown in Table 2.

**Table 2:** Comparison of immune function in NP and NNP

	NP group *x* − ±s (*n* = 54)	NNP group *x* − ±s (*n* = 83)	*t*-test/Wilcoxon	*P*
Lymphocyte subgroup				
CD3+	58.38 (21.33)	59.99 (19.09)	0.48	0.63
CD4+	26.42 (11.44)	27.59 (10.12)	0.65	0.52
CD8+	26.04 (10.61)	26.38 (9.51)	0.20	0.84
CD16 + 56+/CD3+	10.43 (7.24)	12.94 (7.64)	1.96	0.051
CD19+	18.46 (9.36)	17.78 (8.59)	−0.45	0.66
CD4+/CD8+	1.01 (0.56)	1.01 (0.48)	−0.33	0.74
Immunoglobulin				
IgG	7.68 (3.12)	9.53 (2.74)	3.85	0.0002
IgA	2.17 (0.73)	2.33 (0.73)	1.37	0.17
IgM	1.23 (0.49)	1.25 (0.52)	0.32	0.75
IgE	68.15 (136.10)	89.60 (191.20)	−0.92	0.36
Complement				
CH50	50.86 (10.84)	50.47 (11.98)	−0.20	0.85
C3	1.15 (0.22)	1.21 (0.22)	1.45	0.15
C4	0.23 (0.08)	0.22 (0.08)	−0.95	0.34

### Pathological features

#### ISKDC grading score

The results of the ISKDC grading score at onset showed that 35 patients had grade I, 36 had grade II, 48 had grade IIIa, 11 had grade IIIB, and 6 had above grade IV. Grade IIIa was the most common. Among the NP group patients, 5 patients (9.26%) had grade I, 15 (27.78%) had grade II, 34 (62.97%) had above grade III. Different from the NP group patients, most NNP group patients had grade I (37.35%), followed by grade IIIa (32.53%). The results showed a significant difference between the two groups (Table 3) and indicated that the renal pathological tissue damage in the NP group was significantly greater than that in the NNP group.

**Table 3:** Comparison of biopsy findings in NP and NNP

ISKDC grading score	NP group n (%) (*n* = 54)	NNP group n (%) (*n* = 83)	Total n (%)
I	5 (9.26)	31 (37.35)	36 (26.28)
II	15 (27.78)	21 (25.30)	36 (26.28)
IIIa	21 (38.89)	27 (32.53)	48 (35.04)
IIIb	8 (14.81)	3 (3.61)	11 (8.03)
IV a	3 (5.56)	1 (1.20)	4 (2.92)
IV b	0 (0.00)	0 (0.00)	0 (0.00)
Va	0 (0.00)	0 (0.00)	0 (0.00)
Vb	1 (1.85)	0 (0.00)	1 (0.73)
VI	1 (1.85)	0 (0.00)	1 (0.73)

*X* ^*2*^ = 20.58, *P* < 0.01

### Immune complex deposition

Through the kidney mesangial area immunofluorescence sedimentation analysis, 17 cases in the NP group had IgA deposits, 15 had IgA + IgM deposits, and 14 had IgA + IgM + IgG deposits. Most patients had IgA deposits, followed by IgA + IgM deposits. In the NNP group, most patients also had IgA deposits but followed by IgA + IgM + IgG deposits. There was no statistical difference between the two groups in terms of immune complex deposition type (*P* > 0.05). The results are shown in Table 4.

**Table 4:** Comparison of immunofluorescence findings in NP and NNP

Immune-complex deposition	NP group n (%) (*n* = 54)	NNP group *n* (%) (*n* = 83)	Total n (%)
IgA	17 (31.48)	26 (31.33)	43 (31.39)
IgA + IgG	8 (14.81)	18 (21.69)	26 (18.98)
IgA + IgM	15 (27.78)	19 (22.89)	34 (24.82)
IgA + IgG + IgM	14 (25.93)	20 (24.10)	34 (24.82)

*X* ^*2*^ = 1.17,*P* > 0.05

In the study, 63.50% of the children had immune deposits associated with C3 deposition; the rest of the patients did not have C3 deposits. There was no statistical significance between C3 deposition and urine protein quantitative results (*P* > 0.05) (Table 5).

**Table 5:** Comparison of C3 immunofluorescence findings in NP and NNP

	NP group n (%)	NNP group n (%)	Total n (%)
C3 deposits	35 (25.55)	52 (37.96)	72 (63.50)
no C3 deposits	19 (13.87) 31 (22.63)		39 (36.50)

*X* ^*2*^ = 0.0661,*P* > 0.05

### Tubulointerstitial injuries

Of the 137 patients, 91 patients (66.42%) had grade 1, 37 (27.01%) had grade 2, 8 (5.84%) had grade 3, and 1 (0.73%) had grade 4 tubulointerstitial injuries. In both groups, most patients had grade 1, followed by grade 2 injuries. The difference in tubulointerstitial injuries between the two groups had a statistical significance (*P* < 0.01). The results showed that the tubulointerstitial injuries of the NP group were more serious than that of the NNP group. The results are shown in Table 6.

**Table 6:** Comparison of tubulointerstitial findings in NP and NNP

Tubulointerstitial injury grading	NP group n (%)	NNP group n (%)	Total n (%)
grade 1	27 (19.71)	64 (46.72)	91 (66.42)
grade 2	21 (15.33)	16 (11.68)	37 (27.01)
grade 3	5 (3.65)	3 (2.19)	8 (5.84)
grade 4	1 (0.73)	0 (0.00)	1 (0.73)
Total	54 (42.03)	83 (57.97)	137 (100.00)

*X* ^*2*^ = 11.6008, *P* < 0.01

### Nephrotic-range proteinuria in HSPN prognostic analysis

In our study, all patients had a follow-up of 6 months to 5 years, and the mean follow-up was 43.10 ± 16.39 months. Among them, 68.52% had a good outcome (A), 27.78% had mild urinary anomalies (B), only 2 had a remained active renal disease (C), and no patient had an ESRD progression (D).

### Correlation analysis of the clinical classification and prognosis

The clinical classification of the NP group mainly involved hematuria and proteinuria, since there was no case of isolated proteinuria or isolated hematuria. There were 37 patients (68.52%) with a complete remission (A), and only 2 patients (3.7%) had an active renal disease (C). There were clinical manifestations in 6 patients with nephrotic syndrome (11.11%) and mild urinary anomalies (B). One case of radical nephritis developed into minor urinary abnormalities (B). One case of rapidly progressive glomerulonephritis developed into active renal disease (C). As shown in Table 7, the different clinical classifications of the prognostic difference in the NP group had a statistical significance (*P* < 0.01). The more severe the clinical manifestations were, the worse the prognosis was.

**Table 7:** Outcome in relation clinical features at onset

Clinical manifestations	A n (%)	B n (%)	C n (%)	D n (%)
isolated hematuria	0 (0.00)	0 (0.00)	0 (0.00)	0 (0.00)
isolated proteinuria	0 (0.00)	0 (0.00)	0 (0.00)	0 (0.00)
Hematuria and proteinuria	30 (55.56)	8 (14.81)	1 (1.85)	0 (0.00)
radical nephritis	0 (0.00)	1 (1.85)	0 (0.00)	0 (0.00)
Nephrotic syndrome	7 (12.96)	6 (11.11)	0 (0.00)	0 (0.00)
Rapidly progressive glomerulonephritis	0 (0.00)	0 (0.00)	1 (1.85)	0 (0.00)
Total	37 (68.52)	15 (27.78)	2 (3.7)	0 (0.00)

*X* ^*2*^ = 32.3501; *P* < 0.0001

### Pathological grading and prognosis analysis

In the NP group, all patients with grade I had a complete remission (A), 8 (14.81%) with grade II showed a complete remission (A), 6 (11.11%) had mild urinary anomalies (B), and only 1 (1.85%) showed an active renal disease (C). Twenty-five patients (46.29%) with grades III–VI had good outcomes (A), 8 (14.82%) had mild urinary anomalies, and only 1 (1.85%) showed an active renal disease (C). No correlation existed between the pathological grading and prognosis (Table 8).

**Table 8:** Outcome in relation to biopsy findings

Pathological grading	A n (%)	B n (%)	C n (%)	D n (%)
I	4 (7.41)	1 (1.85)	0 (0.00)	0 (0.00)
II	8 (14.81)	6 (11.11)	1 (1.85)	0 (0.00)
IIIa	16 (29.63)	4 (7.41)	1 (1.85)	0 (0.00)
IIIb	4 (7.41)	4 (7.41)	0 (0.00)	0 (0.00)
IV	3 (5.56)	0 (0.00)	0 (0.00)	0 (0.00)
V	1 (1.85)	0 (0.00)	0 (0.00)	0 (0.00)
VI	1 (1.85)	0 (0.00)	0 (0.00)	0 (0.00)
合计	37 (68.52)	15 (27.78)	2 (3.70)	0 (0.00)

*X* ^*2*^ = 7.2936; *P* = 0.8376

### Tubulointerstitial injuries and prognosis analysis

Of 54 patients, 66.67% with grade 1 had a complete remission (A), 8 (29.62%) had a poor prognosis for mild urinary anomalies (B), 13 (65.00%) with grade 2 showed a complete remission (A), and 7 (35.00%) had a poor prognosis for mild urinary anomalies (B). Six patients (85.17%) with grades III–IV had good outcomes (A). Only 1 patient (1.85%) showed an active renal disease (C). No correlation existed between the tubulointerstitial injuries and prognosis (*P* > 0.05). The results are shown in Table 9.

**Table 9:** Outcome in relation to tubulointerstitial findings

Tubulointerstitial injury grading	A n (%)	B n (%)	C n (%)	D n (%)
grade 1	18 (33.33)	8 (14.81)	1 (1.85)	0 (0.00)
grade 2	13 (24.07)	7 (12.96)	0 (0.00)	0 (0.00)
grade 3	5 (9.26)	0 (0.00)	1 (1.85)	0 (0.00)
grade 4	1 (1.85)	0 (0.00)	0 (0.00)	0 (0.00)
Total	37 (68.52)	15 (27.78)	2 (3.70)	0 (0.00)

*X* ^*2*^ = 7.228; *P* = 0.352

### Treatment and prognosis analysis

The therapies of HSPN include steroids, immunosuppressive agents, angiotensin converting enzyme inhibitors (ACE-I) and/or angiotensin receptor blockers (ARB), plasmapheresis, and tonsillectomy. At our institutes, all treatment decisions are based on the clinical and pathological severities of the patient conditions.

In our study, the main treatment in the NP group was steroids combined with mycophenolate mofetil (46.30%). The secondary treatment was methylprednisolone pulse therapy (20–30 mg/kg/d, maximal dose 1 g) for 3 days; steroids combined with mycophenolate mofetil (25.93%) were used thereafter. Thirty-eight patients (70.37%) had a complete remission (A). Only 2 patients (3.70%) showed an active renal disease (C). The results showed that the prognosis of the NP group improved after a timely and early treatment. Further, these therapies had no obvious adverse effects (Table 10).

**Table 10:** Outcome in relation treatment of NP group

Treatment	A n (%)	B n (%)	C n (%)	D n (%)	Total n (%)
steroids	2 (3.70)	1 (1.85)	0 (0.00)	0 (0.00)	3 (5.56)
steroids and GTW	4 (7.41)	1 (1.85)	0 (0.00)	0 (0.00)	5 (9.26)
steroids and MMF	27 (50.00)	10 (18.52)	2 (3.70)	0 (0.00)	39 (72.23)
steroids and CTX	2 (3.70)	2 (3.70)	0 (0.00)	0 (0.00)	4 (7.41)
steroids and CsA	1 (1.85)	0 (0.00)	0 (0.00)	0 (0.00)	1 (1.85)
steroids and FK506	2 (3.70)	0 (0.00)	0 (0.00)	0 (0.00)	2 (3.70)
Total	38 (70.37)	14 (25.93)	2 (3.70)	0 (0.00)	54 (100.00)

*X* ^2^ = 3.26; P > 0.05

*GTW* tripterygium glycosides, *MMF* mycophenolate mofetil, *CTX* cyclophosphamide, *CsA* cyclosporine A, *FK506* tacrolimus

## Discussion

HSPN is a renal disease secondary to HSP; when this occurs, secondary glomerulonephritis, morbidity after nephrotic syndrome, and acute glomerulonephritis commonly develop. An accumulating evidence indicates that protein in the urine is not only a sign of kidney damage but also a crucial indicator for kidney disease progression and a critical prognosis factor [2]. For instance, nephrotic-range proteinuria of HSPN often prompts a poor outcome.

Our study showed that there were significantly more boys than girls, with a ratio of 1.40:1. In the NP group, the ratio was 1.70:1, which is consistent with those of previous reports in the literature [5]. Some studies reported that the risk of HSPN increases with age; in particular, cases of children over 10 years of age were more likely to progress to HSPN [6–8]. The age of the NP group in the present study ranged from 4 to 16 years, and the mean age of onset was 8.39 ± 2.85 years. In the NNP group, the age of onset was 9.21 ± 2.94 years. These results suggest that there exists no significant difference between the 2 groups, which is in contrast to the findings published by a number of other previous studies.

In the analysis of the laboratory test results, the NP group exhibited a significantly lower level of serum Alb and IgG; however, the level of BUN and Cys-C increased significantly. There were few studies on humoral and cellular immunities with various degrees of proteinuria in HSPN [9–11]. We found that the NP group exhibited a significantly lower level of IgG, which agrees with the results of previous reports in the literature [10]. Two viewpoints may explain why the NP group had a lower level of IgG; first, the glomerular filtration membrane electrostatic barrier and/or capillary wall barrier function were impaired, resulting in a large number of IgG lost from the urine; second, it may be related to the abnormal functioning of lymphocytes T and B. The results show that these factors can be used as sensitive biological indicators of the severity of HSPN, which can provide an early and reliable information for its prognosis and treatment.

Nickavar et al. [12] suggested that NP in HSPN had higher pathological grades. Chen et al. [13] in addition to reporting a similar finding also suggested that renal tubulointerstitial lesions positively correlated with the disease. In the present study, the majority of the pathological changes in the NP group involved grade III (53.70%), followed by grade II (27.78%). Consequently, our findings are consistent with those of Chen et al. [13]. The results of the present study suggested that the renal pathological damage in the NP group was significantly greater than that in the NNP group (*P* < 0.05). There is growing concern on renal tubulointerstitial lesions both in China and abroad; however, there is still a lack of large-scale studies. Our study showed that the tubulointerstitial injuries in the NP group were more serious than those in the NNP group, which is in contrast to the findings of a previous study [13].

The main characteristics of the immune complex deposition in HSPN primarily involved IgA deposition, followed by IgG or IgM deposits, and complement components C3, followed by C4 and C1q deposits. Further, the capillary walls have varying degrees of involvement [14]. The present study showed immune complex depositions in NP group (mainly IgA immune deposits, followed by IgA + IgM immune deposits). In the study, 63.5% patients had C3 deposition; the results suggested that the complement immune process was involved in HSPN; however, C3 deposition was not correlated with urinary function (*P* > 0.05).

Several authors have discussed that the prognosis of the patients with HSPN is closely related to its clinical manifestation. In the clinical evaluation of hematuria and/or microscale protein in the urine of children, 5% of the cases developed into chronic kidney failure; 40% of the nephritic syndrome cases progressed to chronic kidney failure. Over 50% of cases that showed both nephrotic syndrome and nephritic syndrome developed into chronic kidney failure. Moreover, even subtle abnormalities in the urine may develop into chronic kidney failure after 10 years [15]. This study shows that patients with hematuria and urinary protein had a good outcome (A) 46.15% of the patients with nephrotic syndrome had mild urinary anomalies and (B) all patients with radical nephritis also had mild urinary anomalies. The description of patients with nephrotic-range proteinuria of HSPN prognosis was closely related to its clinical classification; however, this requires a long-term follow-up study in the future.

We also did not find any difference between the pathological and tubulointerstitial injuries in relation to the outcome, which is in contrast to the findings published by Halling et al. [16], who found that ISKDC grades III–V have a worse outcome than grades I–II. There may be a relationship between small-scale data samples and a reasonable treatment used in a timely manner.

In our study, the choice of treatment for HSPN depended on both the histological and clinical severity of the patient conditions [13, 14, 17]. All patients received ACE-I and/or ARB, and dipyridamole. In the NP group, 72.23% of the patients were treated with steroids and mycophenolate mofetil. After the follow-up of 6 months to 5 years, 38 patients (70.37%) had a complete remission (A), only 2 showed an active renal disease (C), and no cases progressed to ESRD. Our data indicate that a combination therapy, such as steroids with mycophenolate mofetil, is effective for histologically or clinically severe HSPN. However, tripterygium glycosides may affect the reproductive system; this side effect limited its clinical application. In our study, the follow-up period was <5 years; the presence of a poor prognosis may exist over a long period.

## Conclusion

NP in HSPN is a serious condition. The NP group had a significantly lower level of serum Alb and IgG but a significantly increased level of BUN and Cys-C. The biopsy findings were mainly above grade III. The prognosis of the NP group was associated with the clinical classification and improved after a timely and early treatment.

## Abbreviations

Alb: Albumin
APTT: Activated partial prothrombin time
AT-III: Antithrombin III
BUN: Blood urea nitrogen
Cys-C: Cystatin C
eGFR: Estimated glomerular filtration rate
ESRD: End-stage renal disease
FDP: Fibrinogen degradation product
FIB: Fibrinogen
HSPN: Henoch-Schonlein purpura nephritis
Ig: Immunoglobulin
NNP: Non-nephrotic proteinuria
NP: Nephrotic proteinuria
PLT: Platelet
PT: Prothrombin time
Scr: Serum Creatinine
TT: Thrombin time
UA: Uric acid
